# Vitamin B12 Deficiency Presenting With Microangiopathic Hemolytic Anemia

**DOI:** 10.7759/cureus.12600

**Published:** 2021-01-10

**Authors:** Haitham Osman, Turki A Alwasaidi, Abdulqader Al-Hebshi, Najah Almutairi, Hussein Eltabbakh

**Affiliations:** 1 Hematology, Prince Mohammed Bin Abdulaziz Hospital/Ministry of National Guard Health Affairs, Madinah, SAU; 2 Internal Medicine, Taibah University, Madinah, SAU; 3 Pediatric Hematology Oncology, Prince Mohammed Bin Abdulaziz Hospital/Ministry of National Guard Health Affairs, Madinah, SAU; 4 Pediatrics, King Saud Bin Abdulaziz University for Health Sciences, Riyadh, SAU; 5 Internal Medicine, Prince Mohammed Bin Abdulaziz Hospital/Ministry of National Guard Health Affairs, Madinah, SAU

**Keywords:** schistocytes, microangiopathic hemolytic anemia, thrombotic thrombocytopenic purpura, pseudo-tma, vitamin b12 deficiency

## Abstract

Vitamin B12 has essential roles in DNA synthesis, red blood cell development, and neurologic functions. Vitamin B12 deficiency is relatively common, particularly in people aged over 60 years. Among hematological disturbances, microangiopathic hemolytic anemia with thrombocytopenia or so-called pseudo-thrombotic microangiopathy (pseudo-TMA) is a particularly rare but significant clinical complication in patients with vitamin B12 deficiency. We herein describe a case of an elderly patient with pseudo-TMA whose lack of vitamin B12 was misdiagnosed as thrombotic thrombocytopenic purpura (TTP). The patient was admitted as a case of pancytopenia with a hemolytic picture. The initial impression was TTP versus acute promyelocytic leukemia M3. After examination of laboratory tests and bone marrow examination, we deduced that the patient had a B12 deficiency. The condition of the patient improved with B12 replacement. This report should remind physicians to widen their differential diagnoses when patients present with microangiopathic hemolysis or in patients who are not responsive to standard treatments for TTP.

## Introduction

Every vitamin is assigned a specific and unique role in the human body, for instance, “vitamin B12" is one of the most vital vitamins with its unique structure and composition of the mineral cobalt and thus the origin of the name cobalamin. It possesses various roles on different levels, including DNA and red blood cell (RBC) synthesis, in addition to several neurologic functions [1]. The cut-off was defined by the World Health Organization, where cobalamin deficiency was presented as less than 150 pmol/L [2]. During the recent decade, vitamin B12 deficiency has become relatively common, particularly in the population aged over 60 years [3]. A broad range of vitamin B12 deficiency-related clinical evidence has been reported describing clinical severity ladder, ranging from fatigue, anemia, glossitis, and subtle neurologic disturbance in mild-to-moderate cases, to severe hematological abnormalities, severe neurologic manifestations, and/or cardiomyopathy in severe cases [3]. Microangiopathic hemolytic anemia (MAHA) with thrombocytopenia or so-called pseudo-thrombotic microangiopathy (pseudo-TMA) is a particularly significant hematological complication in patients with cobalamin deficiency [4,5]. Damaged RBC membrane can cause intravascular hemolysis, leading to MAHA, as characterized by the appearance of schistocytes (key characteristics of MAHA) [6]. Primary thrombotic microangiopathy syndromes involve serious conditions such as thrombotic thrombocytopenic purpura (TTP), hemolytic uremic syndrome, drug-induced TMA, and complement-mediated TMA. These conditions must be managed and controlled immediately, therefore uncovering the primary etiology including plasmapheresis or monoclonal antibodies that bind complement proteins [7,8]. One of the cobalamin deficiency-TMA features is that patients do not respond to plasma infusion or exchange; the failure to recognize this diagnosis may prompt unnecessary treatments [2]. TTP is a quickly advancing and life-threatening illness that, in past years, featured a classic pentad of MAHA, thrombocytopenia, fever, renal dysfunction, and neurologic abnormalities [9]. Patients with malignancies, as well as those with autoimmune disorders and following solid organ and stem cell transplants, may present with thrombocytopenia and MAHA. In this case, the treatment should be directed at the specific underlying condition [6]. Cobalamin deficiency-induced TMA designates TMA secondary to vitamin B12 deficiency. Usually, cases with pseudo-TMA present with hemolytic anemia, thrombocytopenia, and dysmorphic “fragmented” RBCs. They are often misdiagnosed to have other TMA syndromes and receive unnecessary therapy such as plasmapheresis [1,2]. We herein describe a case of an elderly patient with pseudo-TMA whose lack of vitamin B12 was misdiagnosed with TTP.

## Case presentation

An 84-year-old married male presented to the outpatient clinic for a routine annual check-up. The patient had hypertension and hypothyroidism. He had no change in his bowel habits, no melena or bleeding from any site, no weight loss, no loss of appetite, no fever, no shortness of breath, no headache or other neurologic symptoms, no chest pain, palpitation, or other cardiovascular complaints, and no urinary symptoms. He underwent bowel resection due to intestinal obstruction. Accordingly, he was taking amlodipine 5 mg, thyroxine 75 mcg, and aspirin 81 mg daily. He had no history of smoking, alcohol, or drug use, nor a family history of hematological disease. On physical examination, the patient was fully conscious oriented; his body temperature was 36.8°C, blood pressure was 131/59 mmHg, pulse was 78 beats per minute, respiratory rate was 18 breaths per minute, and oxygen saturation was 98% on room air. He was conscious, oriented, and slightly pale, and had no jaundice or nail changes. His head and neck examination showed no oral ulcers, a normal tongue, no lymphadenopathy, and no peripheral stigmata of chronic liver disease; his jugular venous pressure was not raised, and cardiovascular and respiratory examinations were normal. There were midline abdominal longitudinal and right sub-costal scares; no organomegaly was present. There was a bilateral petechial rash on the anterior aspect of both legs, extending from the knee joint to the ankle joint. His neurologic and musculoskeletal examinations were normal.

His initial laboratory finding (Table 1) revealed pancytopenia with a hemoglobin of 8.2 g/dL (normal: 11.0-14.5 g/dL), platelets of 64 x 10^9^/L (normal: 150-450 x 10^9^/L), WBCs of 1.6 x 10^9^/L (normal: 4-12 x 10^9^/L), absolute neutrophilic count of 0.35 x 10^9^/L (normal: 2-7.5 x 10^9^/L), and absolute lymphocytic count of 0.98 x 10^9^/L (normal: 1.0-4.4 x 10^9^/L).

**Table 1:** Laboratory Investigations Hgb, hemoglobin; Hct, hematocrit; RBC, red blood cells; MCV, mean corpuscular volume; MCH, mean corpuscular hemoglobin; MCV, mean corpuscular volume; WBC, white blood cells; ANC, absolute neutrophilic count; ALC, absolute lymphocytic count; LDH, lactate dehydrogenase

Parameters	Reference values	Results upon admission	Results after one month of treatment
Hgb	11.0-14.5 g/dL	8.2	10
Hct	0.31-0.45 L/L	0.253	0.319
RBC	3.9-5.6 x 10^12^/L	2.74	3.28
MCV	75-89 fL	114	97.1
MCH	25-30 pg	35	30.7
Platelet	150-450 x 10^9^/L	64	273
WBC	4-12 x 10^9^/L	1.6	8
ANC	2-7.5 x 10^9^/L	0.35	4.46
ALC	1-4.4 x 10^9^/L	0.98	2.11
D-Dimer	0.00-0.5 mg/mL	17.44	2.49
Schistocytes	Less than 0.2%	5%	Less than 1%
Reticulocyte	0.5-1.5%	2.22	5.61
Fibrinogen	1.5-4.1 pm/L	1.11	2.5
LDH	125-220 U/L	486	365
Vitamin B12	138-652 pmol/L	61	530

**Figure 1:**
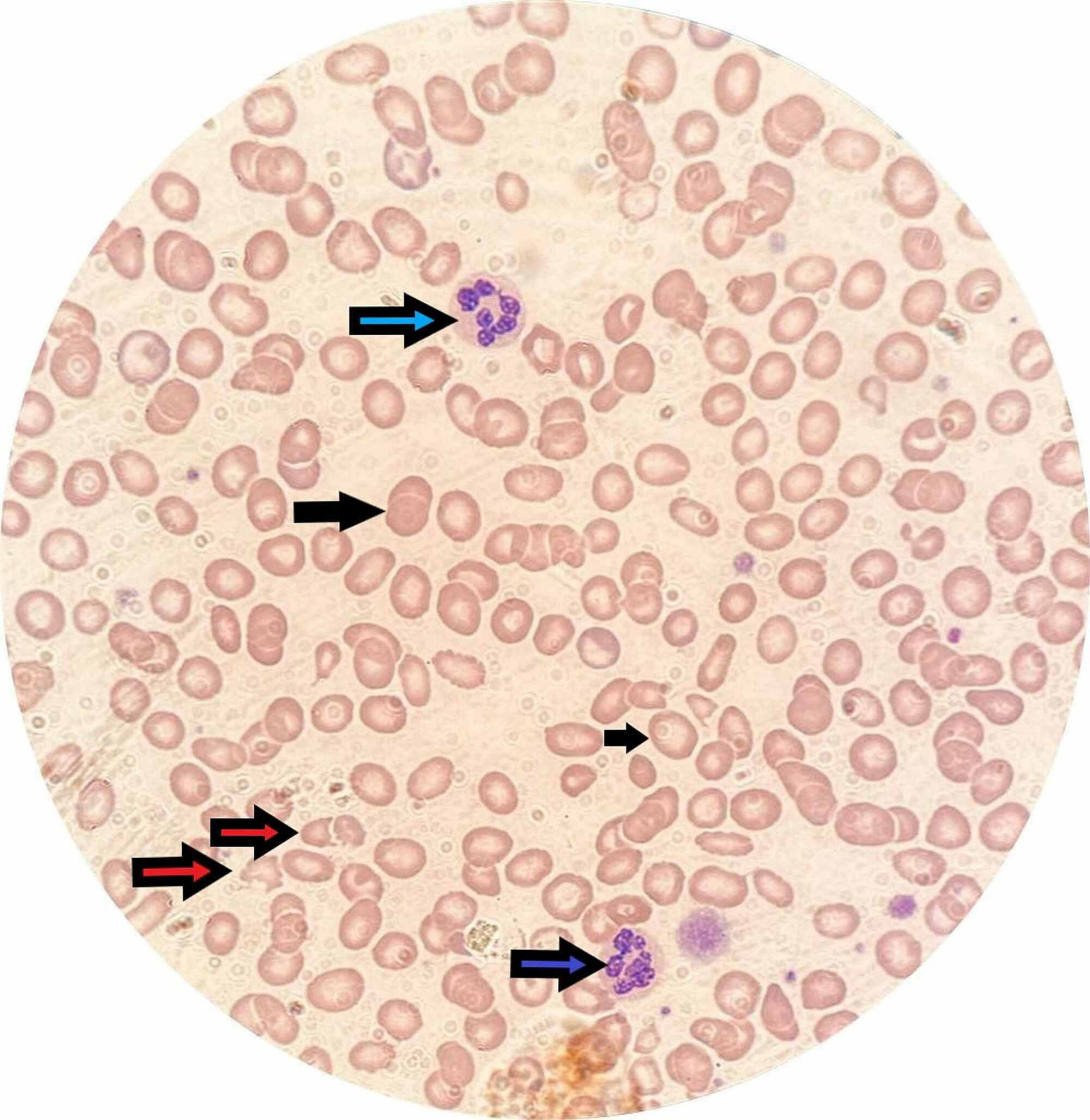
Peripheral blood smear showing schistocytes (red arrows), hypersegmented neutrophils (blue arrows), and macrocytes (black arrows)

The peripheral blood smear showed macrocytosis, with frequent schistocytes of 5%, and hyper-segmented neutrophils (Figure 1). The lactate dehydrogenase (LDH) was 486 U/L (high; normal: 125-220 U/L), total bilirubin was 45 umol/L (high; normal: 0-18 umol/L), haptoglobin was <0.058 g/L (low; normal: 0.36-1.95 g/L), reticulocytes count was 2.2% (normal: 0.5-1.5%); B12, folate levels, viral serology, and autoimmune workup was also conducted. The patient underwent bone marrow aspiration and trephine biopsy, and there was no evidence of acute myeloid leukemia or infiltrative bone marrow.

As per the available laboratory results, the patient was initially diagnosed with MAHA. Though the full pentad of TTP was not fully matched and rest of the laboratory workup results were awaited, he was started empirically on fresh frozen plasma (FFP) transfusion every six hours while ADAMTS13 (a disintegrin and metalloproteinase with a thrombospondin type 1 motif, member 13) was sent for workup. However, after 72 hours of FFP, his blood count did not improve and hemolytic markers were rising. Pan CT was performed showing no hidden malignancy that could trigger MAHA. On the third day of admission, the vitamin B12 level result came back with 61 pmol/L (normal: 138-652 pmol/L); his folate level was normal. He has a normal level of ADAMTS13, normal homocysteine level, and negative anti-intrinsic and anti-parietal cell antibodies.

Finally, we deduced that the patient has severe vitamin B12 deficiency; therefore he was started on intramuscular cyanocobalamin 1,000 mcg once daily for seven days and then changed to once weekly for five weeks and then monthly lifelong.

The patient showed clinical improvement at day 3 of parenteral replacement of vitamin B12; his white blood cells increased to 3.5 x 10^9^/L, hemoglobin to 10 g/dL, and platelet to 100 x 10^9^/L. The schistocytes started to disappear gradually. The complete blood count and vitamin B12 levels were normalized after one month of treatment (Table 1).

## Discussion

This is a detailed case of pseudo-TMA due to extreme vitamin B12 deficiency following bowel resection (terminal ileum). Previous evidence showed that the maintenance of the terminal ileum may protect vitamin B12 retention capacity [10]. Moreover, vitamin B12 deficiency-induced TMA postures a real challenge for professionals managing cases of thrombocytopenia, hemolytic anemia, and schistocytosis. In spite of the fact that the differential diagnosis should aim at ruling out the foremost critical conditions in the first place, estimation of vitamin B12 and methylmalonic level to the current symptomatic board for the assessment of TTP can guide clinicians to appropriate determination and treatment [11]. As mentioned, vitamin B12 plays a significant role in RBC synthesis; therefore, when this compound reaches low levels (cobalamin deficiency), the rigidity of RBC membrane increases and the erythrocyte deformability decreases, causing the lysis of RBCs [12]. Furthermore, cobalamin deficiency not only affects RBCs but also pauses the maturation of all cell lines in the marrow. It can manifest with hemolytic anemia secondary to abnormal erythropoiesis and indirect hyperbilirubinemia, yet it does not often manifest with MAHA. Past literature contains only very few cases of vitamin B12 deficiency-induced MAHA, termed as “pseudo-thrombotic angiopathy.” Others proposed that severe hyperhomocysteinemia combined with vitamin B12 deficiency points to an impressive peripheral blood smear and clinical findings similar to TTP [13].

TTP is the most differential diagnosis of pseudo-TMA that causes a hurdle for the professionals. Although TTP can be deadly without any proper therapy plan, starting plasmapheresis treatment for TTP and vitamin B12 replacement for cobalamin deficiency might be a plausible choice for pseudo-TMA-suspected cases [14]. One of the features that assist in primary diagnosis is that pseudo-TMA does not respond to FFP, which was the case in our report [1].

Vitamin B12 deficiency-related hemolytic anemia may cause hyperbilirubinemia (due to the destruction of RBCs that have not achieved maturation in the marrow), or may also cause extravascular hemolysis, which should not result in microangiopathy [13]. Moreover, the patient in this report presented with frequent appearance of schistocytes on the peripheral blood smear, in addition to elevated bilirubin and LDH levels.

Furthermore, most of our case’s clinical findings aligned with the standard manifestations and laboratory findings of pseudo-TMA previously described by Andrès et al., including hemolytic anemia, hemoglobin (5.1 to 10 g/dL), mean corpuscular volume (112 to 124 fL), platelet count (25,000 to 11,0000/μL) and vitamin B12 level (12 to 70 pg/mL) [15].

## Conclusions

TMA due to vitamin B12 deficiency is a rare condition, yet it must be inspected in all patients with clinical and laboratory manifestations of TTP. Our case highlights the importance of differential diagnoses of vitamin B12 deficiency in cases presenting microangiopathic hemolysis or in cases that are not responsive to standard treatments for TTP.
